# Effect of H_2_O_2_ @CuONPs in the UV Light-Induced Removal of Organic Pollutant Congo Red Dye: Investigation into Mechanism with Additional Biomedical Study

**DOI:** 10.3390/molecules28010410

**Published:** 2023-01-03

**Authors:** Salman Latif, Fahad Abdulaziz, Abdulaziz M. Alanazi, Amal H. Alsehli, Marwah M. Alsowayigh, Abdulaziz A. Alanazi

**Affiliations:** 1 Department of Chemistry, College of Science, University of Ha’il, Ha’il 81451, Saudi Arabia; 2 Department of Chemistry, Faculty of Science, Islamic University of Madinah, Madinah 42351, Saudi Arabia; 3 Chemistry Department, College of Science, Taibah University, Madinah 42353, Saudi Arabia; 4 Chemistry Department, College of Science, King Faisal University, Al-Ahsa 31982, Saudi Arabia; 5 Department of Chemistry, College of Science and Humanities in Al-Kharj, Prince Sattam Bin Abdulaziz University, Al-Kharj 11942, Saudi Arabia

**Keywords:** green synthesis, photo catalyst, degradation pathway, antimicrobial activity, anti-fungal activity

## Abstract

Hazardous dyes in industrial wastewater are an internationally recognized issue for community health. Nanoparticles synthesized through green protocols are a fascinating research field with numerous applications. The current study mainly aimed to investigate the degradation of Congo red (CR) dye under UV light in the presence of H_2_O_2_ and the photocatalytic activity of copper oxide nanoparticles (CuONPs). For CuONP formation, *Citrus maxima* extract contains a high number of phytochemical constituents. The size of CuONPs ranges between 25 and 90 nm. The photocatalytic activity of CuONPs with the addition of H_2_O_2_ was observed and analyzed under UV light to eliminate CR dye. The UV light caused the decomposition of H_2_O_2_, which produced ·OH radicals. The results revealed a significant increment in dye degradation during the presence of H_2_O_2_. The effect of concentration on the degradation of the CR dye was also studied. The degradation pathway of organic pollutants was reputable from the hydroxy radical medicated degradation of CR. Advanced Oxidation Treatment depends on the in situ production of reactive ·OH species and is presented as the most effective procedure for decontamination. The biological activity of CuONPs was evaluated against *Escherichia coli Bacillus subtillis*, *Staphylococcus aureus*, *Shigella flexenari*, *Acinetobacter Klebsiella pneumonia*, *Salmonella typhi* and *Micrococcus luteus.* The newly synthesised nanomaterials showed strong inhibition activity against *Escherichia coli* (45%), *Bacillus subtilis* (42%) and *Acinetobacter species* (25%). The activity of CuONPs was also investigated against different fungus species such as: *Aspergillus flavus*, *A. niger*, *Candida glabrata*, *T. longifusus*, *M. Canis*, *C. glabrata* and showed a good inhibition zone against *Candida glabrata* 75%, *Aspergillus flavus 68%*, T. *longifusus* 60%. The materials showed good activity against *C. glaberata*, *A. flavus* and *T. longifusus.* Furthermore, CuONPs were tested for antioxidant properties using 2, 2 diphenyl-1-picrylhydrazyl) (DPPH).

## 1. Introduction 

Industrial effluents from factories comprise significant amounts of pigments and other organic pollutants that are highly stable; however, they can cause severe health issues (kidney disorders, cancer, etc.) and are challenging to remediate [1,2,3,4,5,6,7]. Nanoparticles (NPs) have gained significant attention over time due to their unique properties and high surface-to-volume ratios [1,8,9]. Nanotechnology is an ever-growing discipline dedicated to synthesizing and promoting the use of NPs [10,11,12,13,14]. The antimicrobial properties of NPs such as chitosan, AgNPs, photocatalytic TiO_2_ and other carbon nanotubes (CNT) have already been reported [15,16,17]. Nanotechnology is widely used for developing effective catalysts with high biochemical activity to control microbial infections and diseases [18,19,20,21]. Nanotechnology is a multidisciplinary field of engineering [22,23,24] medicine with a wide range of applications for molecular imaging, diagnosis and targeted therapy [25]. This new field is making a substantial contribution to medical science as an innovative and useful tool [24,25,26,27]. The use of nanomaterials for diagnostic and screening purposes [27,28,29,30,31], the development of artificial proteins, receptors, DNA and protein sequencing with nano-pores and nano-sprays [32,33,34], novel drug delivery systems (DDS), gene therapy and tissue-engineering applications [13] are all examples of nanomedicine. Water pollution by hazardous chemicals is threatening human life. Many pigments (dyes) released from industries are not degradable [35,36,37]. Many sectors use dyes as a primary chemical, such as the textile, food and cosmetics industries. Their by-products are directly disposed of in water bodies [3,36]. 

To disinfect pollutants, reverse osmosis and attachment to activated carbons are the most frequently used processes [29]. There are several methods to treat wastewater, but they are not cost-efficient and are less reliable [13]. Green synthesis is a good process for metal nanoparticle synthesis that plays a significant role in wastewater treatment. Nanoparticles are vital in the medical field; dyes and inorganic and organic contaminants are eliminated compared to their bulk structure [13]. Because of their superior applications and multi-purpose properties such as sensors [1], memory devices [38], photo catalysis [39], drug delivery [24], catalysis [23], magnetic resonance imaging [40,41] in the treatment of cancer cells [42,43] and CoAl_2_O_4_ nanocrystals [44,45], there has been a growing interest in synthesizing nanoparticles of Fe, Co and Ni in recent years. Nickel nanoparticles play important electrocatalytic, cytotoxic, antimicrobial [46], sensor, photo- and magmatic device roles [13]. Different methods can be used to synthesize CuONPs, as they are extremely hard materials that lack the ductility and malleability of bulk copper. Plant-mediated NP synthesis plays an important role in the reduction of metal ion. Plant extracts are used as a powerful redox agent [47,48,49,50,51]. The present research is focused on investigating the photocatalytic, CuONPs/H_2_O_2_ effect, advanced oxidation process and toxicity of water-soluble dye and its daughter products (DPs). Green synthesized CuONPs were used with H_2_O_2_ to enhance catalytic activity. Further, the antibacterial and anti-fungal activities were studied against different bacteria and fungi [52,53,54].

## 2. Results and Discussion

### 2.1. Ultraviolet-Visible Spectroscopic Analysis

**Figure 1:**
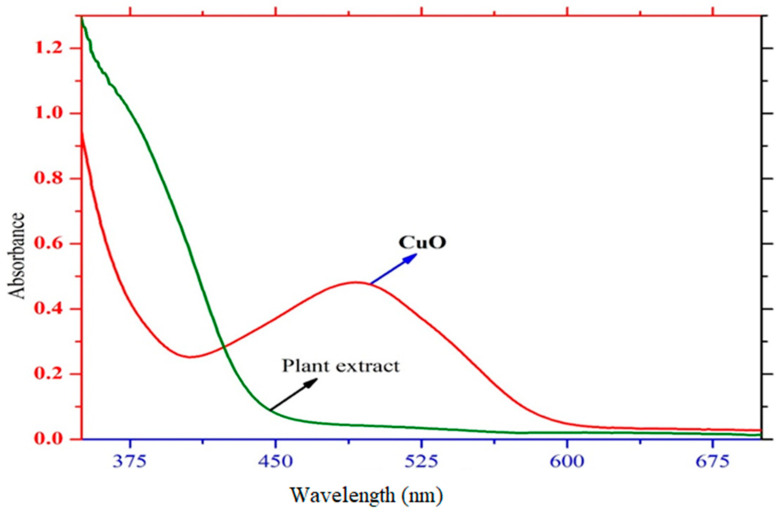
UV/Vis spectrum of CuONPs.

The wavelength for the maximum CuONP formation SPR was determined through a UV/vis system as shown in Figure 1 [5,24]. The single plasmonic band shows the spherical shape of the CuONPs [5,25]. The valence electron excites from HOMO to LUMO through UV/Vis spectroscopy. The electrons move from a lower energy level to a higher energy level (n-σ*, σ-σ*, n-π* and π-π*) when measured under ultraviolet spectroscopy and gaps of energy formed among HOMO and LUMO and transition states are determined by absorption. For the validation of the CuONP synthesis, UV/visible spectra are conducted. The biosynthesis of the CuONPs is confirmed by the absorption spectrum (Figure 1). A UV/Vis spectrometer is used to confirm the synthesis of CuONPs by measuring the range of 470–475 nm. The presence of CuONPs in the reaction mixture clearly illustrates the SPR peak at 389 nm [16,38]. The low-intensity absorption of the plant extract indicates the formation of a minor quantity of CuONPs. The broadness and the concentration of the absorption band depend on time. The Plasmon peak demonstrates high intensity using 20 mL of the extract. However, the particle size also increases with the increment in plant extracts [13]. The % of hydroxyl and bioactive compounds affect the plant materials in particle size and shape for NP synthesis and Cu reduction. These -OH and bioactive compounds cause the reduction of metallic ions [14].

### 2.2. Fourier Transform Infrared Spectroscopy Analysis

**Figure 2:**
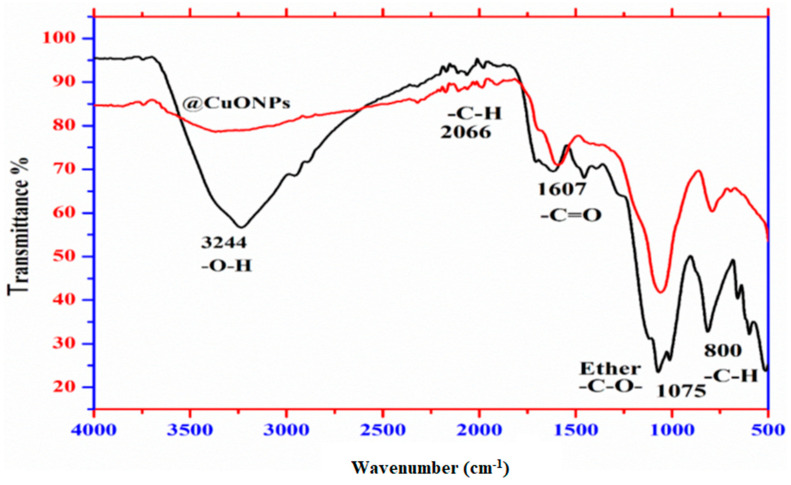
The FTIR spectrum of CuONPs.

To assist in CuONP stabilization and capping, the FT-IR spectrum expresses the possible organic complex adherent [13,25,38]. This method mainly depends on the absorption of small molecules. It also measures the functional groups and evaluates the atom’s vibrations and the compound’s structure in a molecule [46,55]. A 4000–400 cm^−1^ wavenumber region of the IR absorption bands was attained in this process [56]. The absorption peaks’ ranges at 3234, 2060, 1707, 1075 and 800 cm^−1^ were demonstrated by FTIR spectroscopy, Figure 2. The absorption bands at 2066 and 1607 cm^−1^ appear because of the aldehyde carbon-hydrogen stretching and the aromatic carbon and carbon vibration, respectively [57]. Due to the ethylene group, the stretching vibrations responsible for the 1075 cm^−1^ band and the absorption band at 800 cm^−1^ are ascribed to the C-O carboxylic acid and –C-O-C- ether stretching vibrations. The absorption band at 1040 cm^−1^ is mainly due to the stretching vibration of the C-H bond [32].

### 2.3. X-ray Diffraction

**Figure 3:**
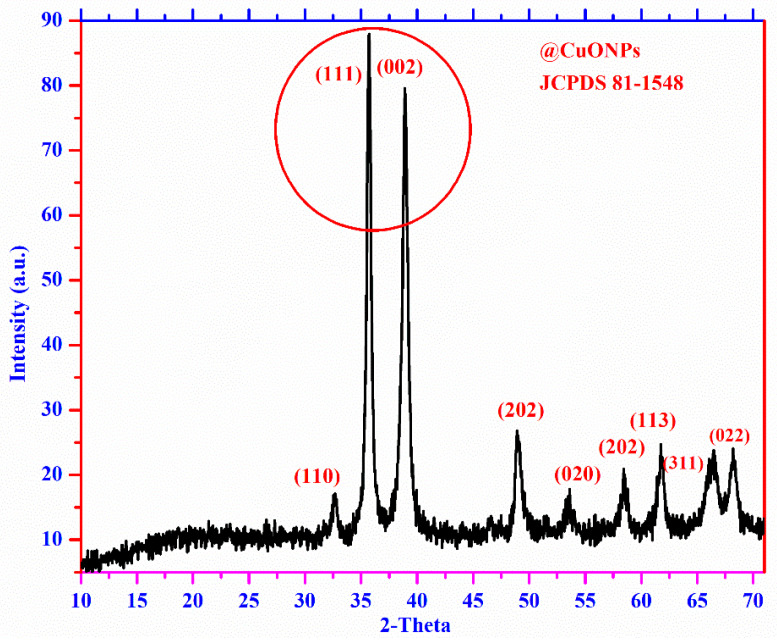
The XRD spectrum of CuONPs.

The crystalline structure of the CuONPs was examined by advanced physical technique XRD, as shown in Figure 3 [27]. An analytical and nondestructive X-ray diffraction method was used to characterize various crystalline matter structures and compositions. The diffraction of the X-rays occurs in the lattice planes at a typical angle in a sample. Atomic distribution measures the peak intensities within a lattice [58,59]. X-ray diffraction analysis at 10°–70° values confirmed that the CuONPs were crystalline [59]. The CuONPs were identified through the presence of a peak at 2*θ* = 36.21 [14,57]. At lattice planes of (110), (111), (002), (112), (202), (202), (020), (202), (113), (311) and (022) (JCPDS 48-1548) [59] the XRD pattern determined the highest purity of the CuONPs. In cases where other peaks were not assigned and there was capping of the biogenic substances at the CuONP surface using the Scherer formula, the shapes and sizes of particles were calculated according to Equation (1) [38,59].

(1) D=0.94 λ /ßcos θ

During analysis, D denotes crystalline; whereas X-ray represents λ, the full width at half maximum is expressed as ß, and the diffraction angle is expressed as *θ*.

### 2.4. Scanning Electron Microscopy (SEM)

**Figure 4:**
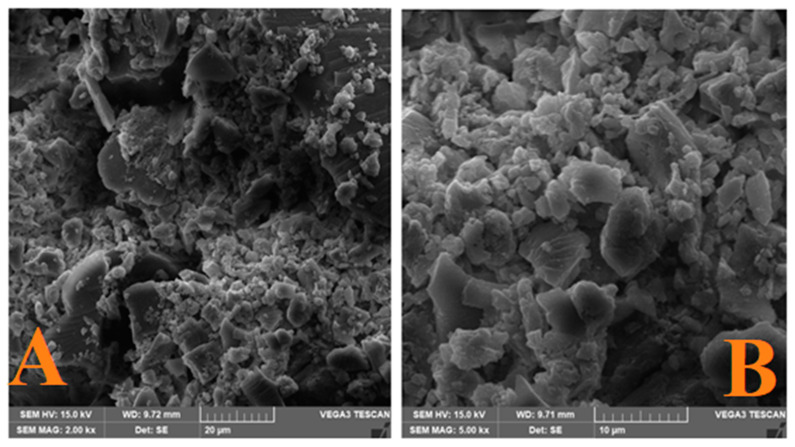
The SEM Images of CuONPs at (**A**) 20 µm and (**B**) 10 µm.

The Scanning Electron Microscopy (SEM) technique is mainly used to measure the surface activity of particles. The SEM of the green synthesised CuONPs of different scales is shown in Figure 4A,B [27], which provides detailed information on the sample surface by tracing the specimen in a raster with an *en* stream of light. The activity increases as the size remains small and the surface area is larger. Hence, due to the existence of colorants in the contaminated water, the particles showed good adsorption properties [5,13].

### 2.5. High-Resolution Transmission Electron Microscopy (HRTEM)

**Figure 5:**
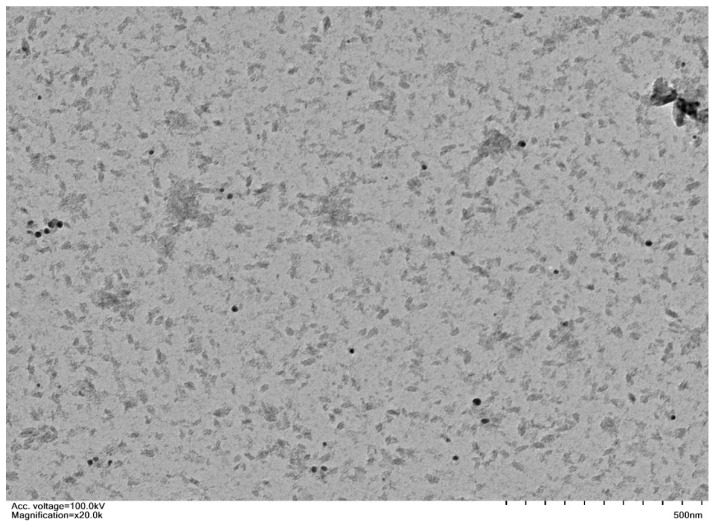
The HRTEM Images of CuONPs.

The results showed that CuONPs are spherical and exhibit a small size and are not aggregated but well dispersed, which is closely associated with SPR absorption in UV spectra, as shown in Figure 5. Substantial activity in various fields was shown by nanoparticles having a larger surface area. In the chemo, electrochemistry and medical fields, well-dispersed and small-size NPs show significant activity [5,60,61].

### 2.6. Energy-Dispersive X-ray Spectroscopy (EDX)

**Figure 6:**
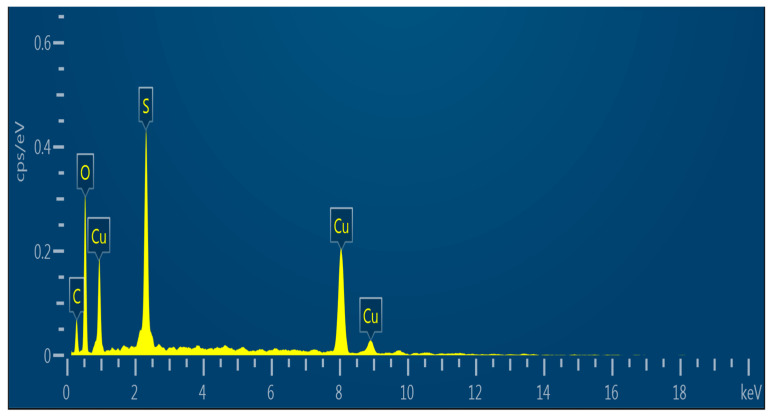
The EDX spectrum of CuONPs.

The elemental conformation and analysis of CuONPs were confirmed by EDX, as shown in Figure 6. The arrangement of the EDX demonstrated that the Cu solution reduction with *Citrus maxima* extracts yielded CuONPs in crystalline form.

**Figure 7:**
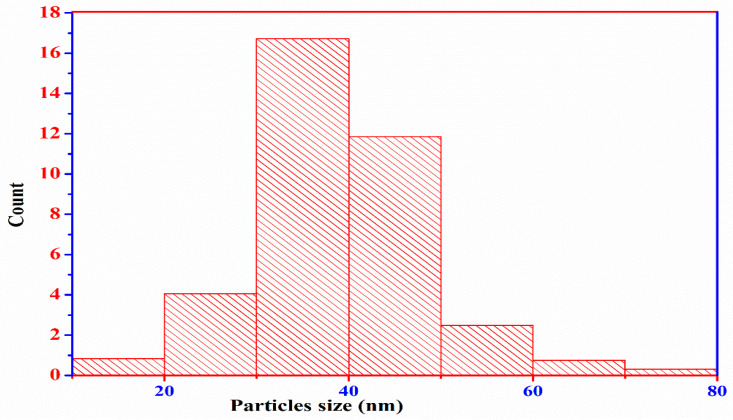
Histogram spectrum of CuONPs.

To determine the particle size of the @CuONPs, a histogram was used. For the determination of particle size, ImageJ software was used [14], as shown in Figure 7. The histogram graph gives the information that the @CuONPs were of a small size and well dispersed. The particle size of the @CuONPs is calculated in the range of 0.6–85 nm with an average size of up to 35 nm.

### 2.7. Antibacterial and Antifungal Activities of CuONPs

The synthesised CuONPs have significant antimicrobial properties against Escherichia coli Bacillus subtillis, Staphylococcus aureus, Shigella flexenari, Acinetobacter Klebsiella pneumonia, Salmonella typhi and Micrococcus luteus [62]. The respective inhibition zones are as 45, 42, 25, 34, 35 and 11 mm compared to the standard 45 mm. The biogenic CuONPs have significant antibacterial activity because of their smaller spherical size, with high dispersion without aggregation. As a consequence of the decomposition of the CuONPs, Cu^2+^ ion concentration is increased [63]. The antibacterial activity of the CuONPs is increased due to the deposition of radicals at the surface of the CuONPs that cause damage to the membrane. Moreover, CuONPs entering and depositing in the bacterial cytoplasm cause cell death [64]. The Fenton reagent-generated ^•^OH radical increases the antimicrobial activities of the CuONPs and causes damage to the DNA [64]. Superoxide reduces Cu^2+^ and releases from ferritin protein according to Equation (2). Table 1 shows the zone of inhibition against different bacteria.

(2) $${\;O}_{2}^{.-}+{\mathrm{Cu}}^{2+}\to {\;\mathrm{Cu}}^{0}+{\;O}_{2}$$

Reactive Cu^2+^ creates reactive [O] species, as shown in Equation (3).

(3) $${\mathrm{Cu}}^{2+}+{\;H}^{+}+{\;H}_{2}O_{2\;}\to {\mathrm{Cu}}^{0}+{\;\mathrm{HO}}^{•}+H_{2}$$

Antifungal activities were studied against *Aspergillus flavus*, *Aspergillus fumigatus*, *Asper gillusniger* and the reference drug *Terbinafine* [65]. To investigate the linear growth of the fungus strain, useable tubes were incubated at room temperature for one week. Mycelia growth inhibition was measured with the fungal growth area and was shown in percent inhibition [64,65,66,67]. The CuONPs showed significant properties against these fungi. Table 2 shows the results of the anti-fungal activities.

### 2.8. Antioxidant Activity

The CuONPs showed significant scavenging properties against DPPH as shown in Table 3. The antioxidant activity mainly relies on the reducing influence of DPPH [68]. The CuONPs showed effective free radical inhibition at 25, 50, 75, 100 and IC_50_ μg/mL was measured with IC_50_ values of 55.02 ± 0.05, 65.7 ± 0.15 and 69.01 ± 0.21 (uM/mL) [69]. The antioxidant activities increase with the increase in concentration of CuONPs. The smaller particle size and large surface area have significant antioxidant activity. The antiradical activity mainly relies on the DPPH-reducing power [69]. Many antioxidants have overcome the action of free radicals [70] to improve the body’s immune system [70,71].

### 2.9. Dilapidation of CR in the Presence of H_2_O_2_

**Figure 8:**
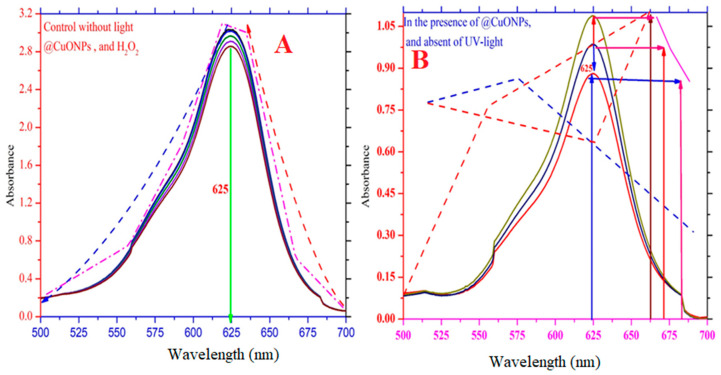
Photo-degradation of Congo red dye: (**A**) Effect of light control; (**B**) Effect of catalyst.

The photodegradation of the CR dye was studied under UV light with CuONPs and UV light with H_2_O_2_ to investigate the effect of H_2_O_2_ in the removal of the CR dye, as shown in Figure 8A,B. During the reaction of UV/H_2_O_2,_ hydroxy free radicals are generated, which play important role in the elimination of the CR dye [72,73,74]. The UV/H_2_O_2_ enhanced the photodegradation of the CR in comparison with UV radioactivity. With the variation in H_2_O_2_ directly related to the degradation rate of dyes during the process, maximum degradation was achieved [73]. During the Fenton-type reaction, ^●^OH is generated and acts as a strong oxidizing agent as an electron scavenger [73,74,75].

### 2.10. Effect of H_2_O_2_ Concentration in the Degradation of CR Dyes

**Figure 9:**
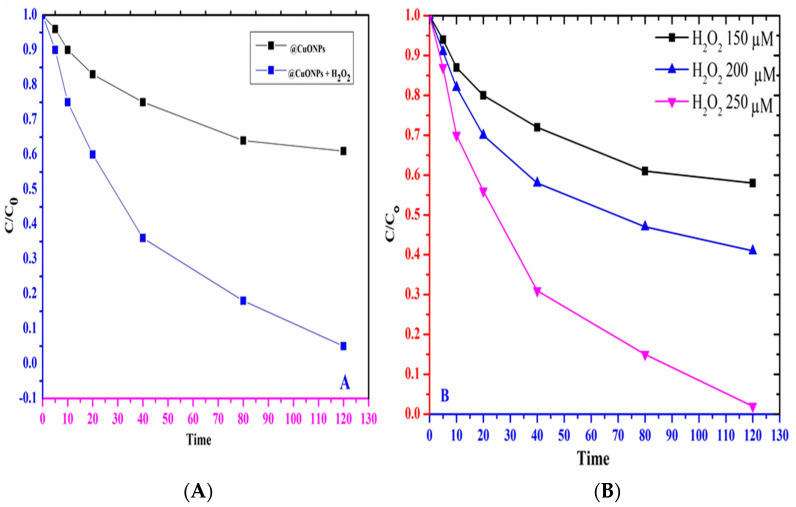
(**A**) Effect and removal of CR dye by UV/ H_2_O_2_; (**B**) Effect of H_2_O_2_ concentration in the degradation of CR dyes.

The concentration effect of UV/H_2_O_2_ was investigated in the degradation of the CR dye. The reports showed that UV/H_2_O_2_ plays a vital role in CR dye removal. The effect of the concertation of the H_2_O_2_ in the removal of CR dyes is shown in Figure 9A,B. The production of the ^●^OH radical under UV light stimulates the rate of reaction. The effect of the ^●^OH radical in the removal of CR dyes was studied using different concentrations of H_2_O_2_ (i.e., 150 µM to 250 µM), while the CR concentration was kept constant. The results showed that the increase in H_2_O_2_ dosage under UV light caused a significant removal of CR dye. It is reported [74] that, with the increase in oxidant concentration, the rate of ·OH radical production increases, which might help remove organic pollutants.

### 2.11. Photocatalytic Activity of CuONPs

**Figure 10:**
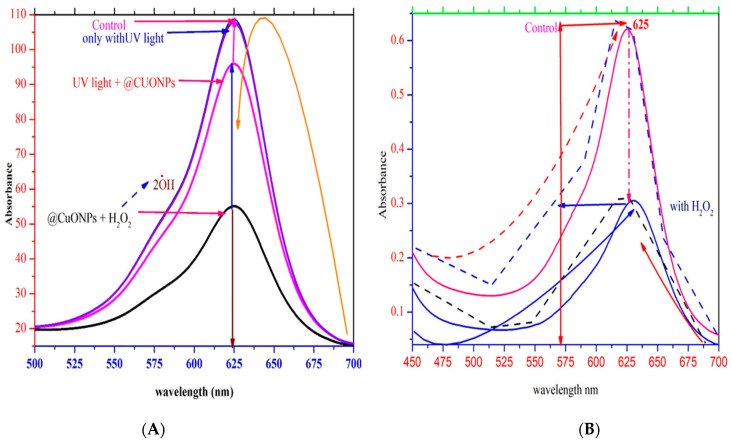
Photo dilapidation of CR dye: (**A**) Effect of light control, light effect, light and @CuONPs and @CuONPs and H_2_O_2_ effect; (**B**) Effect and comparison of H_2_O_2_ on the degradation of Congo red dye.

**Scheme 1:**
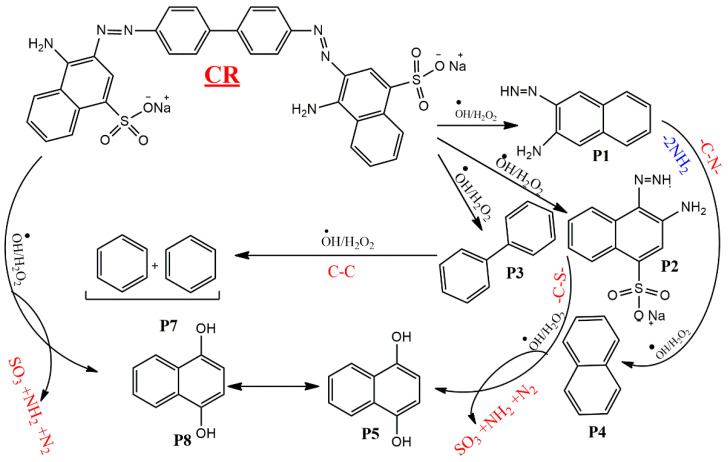
Proposed mechanism of degradation of Congo red dye.

The as-synthesized CuONPs showed significant photocatalytic activity [75,76]. Different types of organic pollutants and toxic materials are present in industrial effluents, which on release to water bodies cause many adverse environmental impacts [77]. Congo red dye shows resistance to heat and light for their removal, because of having a bulky group structure [77]. The LC analysis of Aqueous Congo red dye solution treated with UV/visible light proposed the formation of 8 degradation products (DPs). The photocatalytic activity using UV light increases with the increase in catalyst concertation and H_2_O_2_ dosage up to some limits [78,79]. The UV light shows excellent results in the degradation of CR [80,81,82]. It is suggested that, at the surface of CuONPs, CR adsorbs UV light and reacts with ^•^OH free radical, causing the degradation of CR dyes as shown in Figure 10. The mechanism of the degradation of CR by CuONPs under UV light follows: (i) CR absorbed physically at the CuONPs; (ii) the ^•^OH radical generated during the Fenton-type reaction initiating the degradation of CR under radiation. The proposed reductive and oxidative mechanistic degradation pathway of the CR dyes is represented in Scheme 1. During the heterogeneous photocatalytic degradation, the formation of a positive hole (h^+^_vb_) takes place. The catalytic reaction under UV light is shown in reaction **7**. During this process, the valence electron jumps to an excited and higher energy level from the valance band [80]. In the degradation process, h^+^_vb_ react with hydroxy, hydrogen and water. Due to the high reactivity of the ^●^OH toward CR dyes, degradation occur as shown in Equations (4)–(11) [81].

(4) $$\mathrm{CuO}+\mathrm{hv}\;\left(\mathrm{UV}\right)\to \mathrm{CuO}\left(e_{\mathrm{CB}}^{-}+h_{\mathrm{VB}}^{+}\right)$$

(5) $$\;\mathrm{CuO}\;\left(h_{\mathrm{VB}}\right)+H202\to \mathrm{CuO}+{}^{•}H+{}^{•}\mathrm{OH}$$

(6) $$\;\mathrm{CuO}(h_{\mathrm{vb}}^{+}){}^{•}+{}^{•}\mathrm{OH}\to \mathrm{CuO}+{}^{•}\mathrm{OH}$$

(7) $$\;\mathrm{CuO}\left(e_{\mathrm{CB}}^{-}\right)+{\;O}_{2\;}\to \mathrm{CuO}+{\;O}_{2}^{.-}\;$$

(8) $$O_{2}^{.-}+{\;H}^{+}\to {\mathrm{HO}{}^{•}}_{2}$$

(9) $$\mathrm{Dye}+{\;\mathrm{HO}}^{{}^{•}}\to \mathrm{Degradation}\;\mathrm{products}\;$$

(10) $$\mathrm{Dye}+{\;h}_{\mathrm{VB}+\to \mathrm{Oxidation}\;\mathrm{Products}\;}$$

(11) $$\mathrm{Dye}+{\;e}^{-\mathrm{CB}}\;\to \mathrm{Reduction}\;\mathrm{Products}\;$$

The degradation of the dye was 25% through CuONPs in the absence of UV as compared to the presence of UV light (91%), as shown in Figure 10A,B. The catalyst CuONP concentration ranged from 0.5 to 1.5 g/L. However, at a catalyst concentration (1.0 g/L) of about (91%), the removal of the CR dye occurs. The degradation of the CR dye was studied previously [72]; here, the effect of H_2_O_2_ along with catalyst CuONPs in the removal of CR was studied. The result shows that with the addition of H_2_O_2_, with CuONPs the catalytic efficiency increases much more than without H_2_O_2_. Due to the creation of the ^●^OH radical, the degradation of the dye increases because the hydroxy radical oxidative reagent causes degradation. The higher dye degradation with CuONPs is because of the ^●^OH radical generated during UV light. The ^●^OH radicals have a high redox potential (2.8 V), are extremely reactive oxidants and react rapidly at high rates with the target contaminants [55,72]. Tert-butanol was used as a scavenger for % ^●^OH and the production of % ^●^OH. The removal effectiveness inhibition of the dye in the alcohol’s presence recommends the creation of % ^●^OH from CuONPs; photo light has a high contribution in the dye degradation. The production of various degradation products (P) results from the degradation of the dye (P1) started by % ^●^OH [14,25]. Because of the significant acidic behavior of the H at P1, C-35 illustrates great reactivity toward the hydroxyl radical and is therefore abstracted easily. As illustrated in Scheme 1, P2 upon functional group interconversion is transformed into P3. Moreover, the % ^●^OH reaction with P3 results in the transformation of P3 into P4 through the removal of -SO_3_H from C-54 [72]. A further oxidation process occurs at the C=C group change into P5. P5 is changed to high reactive species P6 when the hydroxyl radical abstracts -H from C-50. By eliminating many functional groups, P6 is converted to P7 by participating in the % ^●^OH group [55,72]. Furthermore, the reaction paths in the solution and gas phases were evaluated on the reaction time scale [55]. By the adduct of –O-O-, the generalization of -H from the –N-N- bond from hydrazones [72] and based on (FGI) P7 is converted to P8. The CR dyes are degraded into smaller molecules as the reaction continues and does not stop. Scheme 1 represents the complete process of the degradation of CR. It shows that the morphology of the CuONPs is significant in the photocatalytic activity. Without CuONPs, there was no progress in the reaction [72].

## 3. Materials and Methods

### 3.1. Preparation of Plant Extract from Citrus maxima

The *Citrus maxima* plant was collected from the field and was washed several times with clean water to remove the dust material. The plant extract contains phenolic constituents and other bioactive organic compounds. The plant biomass was kept under shade to dry at room temperature and ground into a fine powder [25]. To obtain the plant extract, 20 g of plant powder was soaked in 150 mL DW and heated at 50 °C for 12 h with constant stirring. After filtration, the plant extract was used for the synthesis of CuONPs [5].

### 3.2. Synthesis of CuONPs

**Scheme 2:**
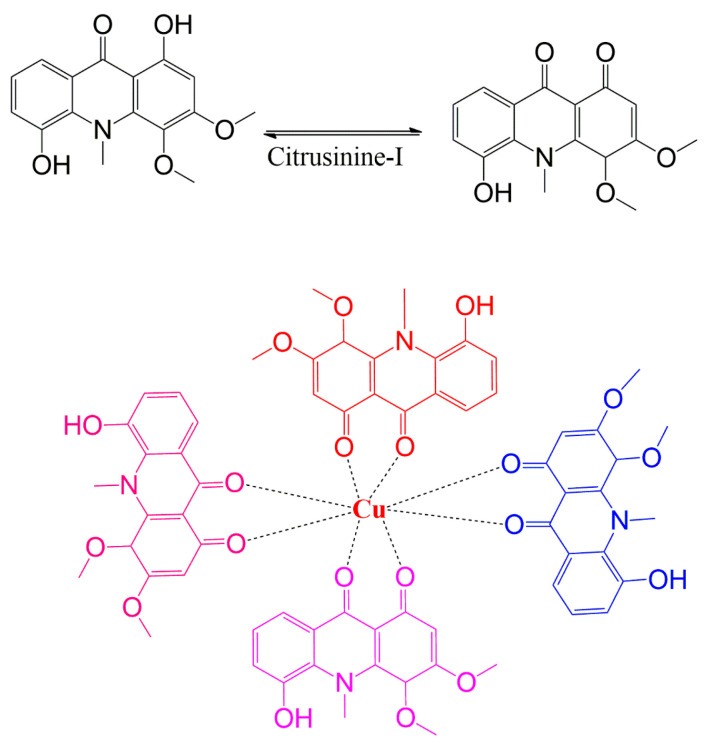
Schematic and proposed mechanistic reduction of Cu^2+^ into zero valent ion.

*Citrus maxima* plant extract (15 mL) was mixed with 50 mL of 6 × 10^−3^ solution of CuSO_4_ to synthesize CuONPs [1,13,24,25] at 35–40 °C with continuous stirring. During the stirring process, the dark color of the solution changed to a greenish color. UV/Visible spectroscopy was used to investigate the plasmonic peak and synthesis process of CuONPs [14]. After the UV confirmation, the suspension was centrifuged for 15 min at 5000 rpm. The suspension was dried under a vacuum and CuONPs were isolated [38]. The reduction of ${\mathrm{Cu}}^{2+}$ to ${\mathrm{Cu}}^{0}$ was caused by the phytochemicals of *C. maxima*. The phenolic constituents in plant extract, especially alcoholic compounds, caused the reduction and stabilization of metallic ions. The phenolic and alcoholic constituents of the medicinal plant oxidized rapidly due to ${\mathrm{Cu}}^{2+}$ through autoxidation. The autoxidation of ${\mathrm{Cu}}^{2+}$ to Cu^0^ through phenolic constituents is represented in Scheme 2**.**

### 3.3. Antibacterial Response of CuONPs

The antibacterial activity of *CuONPs* was screened through AWD (Agar Well Diffusion) method. All the bacterial strains were grown using the nutritive broth (Oxide) at 37 °C in the incubator for 24 h until the level of turbidity touched 0.5 (using the McFarland standard) [5]. For streaking, inocula of *Escherichia coli*, *Bacillus subtillis*, *Staphylococcus aureus*, *Shigella flexenari*, *Acinetobacter Klebsiella pneumonia*, *Salmonella typhi* and *Micrococcus luteus* using Muller Hinton Agar were spread in PD (Petri dishes) to confirm the strained growth. Using cork, 8 mm wells were bored in the Petri plates [24]. Biogenic CuONPs were placed in plates and wells at 25 °C. After 24 h growth at 37 °C, the subsequent region’s diameter of inhibition was determined. The biosynthesized CuONPs exhibited substantial activity, which was confirmed by studies from the literature [1,5,13,24,38]. The experiment was performed in triplicate and the results obtained were expressed as means ± SD.

### 3.4. ROS Generation by CuONPs

To identify reactive oxygen class (ROS) (^•^OH) within the cells, 2, 7-dichlorodihydrofluorescein diacetate (DCFH-DA) was used, which is a moderately useful indicator. The biological activity of CuONPs was investigated using several bacterial strains, *Escherichia coli*, *Bacillus subtillis*, *Staphylococcus aureus*, *Shigella flexenari*, *Acinetobacter Klebsiella pneumonia* and *Salmonella typhi* species. The strains were incubated at 300 rpm for 180 min and 10,000 for 5 min. The suspension of pellets in 1 mL of buffer solution was preserved with one mM (DCFH-DA) reagent for about 40 min. The phosphate buffer solution was used in the cells several times to diminish the excess of dyes from the surface of the cells [14,25,38].

### 3.5. Antifungal Activity of CuONPs

Sabouraud dextrose agar was used to calculate the antifungal properties of biosynthesized CuONPs against various species of fungi, i.e., *Aspergillus flavus*, *A. niger*, *Candida glaberata*, *T. longifusus*, *M. Canis*, *C. glaberata* at 25 ± 1 °C incubation temperature for 7 days [14]. The dispersion of nanoparticles at 100 μg/mL concentration in medium was confirmed by ultrasonic treatment, as they were included in pure dimethyl sulfoxide (DMSO). To ensure sterile conditions, sabouraud dextrose agar was used to pack the test tubes and then exposed to an autoclave. After autoclaving, 100 μL of nanoparticle suspension was added to test tubes and retained in an inclined position. To detect the linear growth of fungus strains, 5 mm of the fungal colony was retained at the slant end, and all test tubes were incubated at 25 ± 1 °C for 7 days [24]. Using *Terbinafineas*, a fungicide reference, Equation (12) was applied to estimate mycelial growth (%) from the fungal growth area (cm^2^) [15].

(12) (%) I=(Gc−Gt|Gc)×100

In Equation (1), the growth of mycelia (control) is represented by Gc and the mycelia growth with the treatment of CuONPs is represented by Gt.

### 3.6. Free Radical Scavenging Assay (DPPH)

The antioxidant activity of the synthesized CuONPs was determined using DPPH [5,13,14,24,27,38]. Optima Sp-300 (Optima, Tokyo, Japan) displayed the maximum absorption at 517 nm for Free DPPH nm and decreased dramatically with antioxidants. The DPPH solution of various concentrations (25–500) was made with methanol (CH_3_OH) with constant stirring and ascorbic acid was used as standard. The control was DPPH methanol without a sample. Antioxidant activity was calculated using Equation (13).

(13) $$\%\;\mathrm{Inhibition}=(\frac{{}_{Ac-At}}{Ac})\;\times \;100$$

### 3.7. Photocatalytic Activity

The photocatalytic behavior of CuONPs under UV light was examined for CR dye removal [14]. The CR dye solution (15 mL) with 10 mg of CuONPs was mixed to prepare the reaction mixture. The solution was magnetically stirred under UV light for some time before being centrifuged to eliminate the catalyst [29]. The Hg lamp of lower pressure with monochromatic emanation at 245 nm was used as a light source. To find out the photocatalytic degradation behavior of CuONPs, CR dye solution sample was taken after five min to study the absorbance through UV light. For the establishment of equilibrium of CuONPs in the mixture, the suspending solution was gently stirred for thirty minutes in dark media instead of being exposed to light. The photodegradation of CR was studied at different concentrations of CuONPs over time (5–25 min). The CR degradation (%) was calculated with Equation (14).

(14) $$\%\;\mathrm{Degradation}=(\frac{{}_{Ac-At}}{Ac})\;\times \;100$$

where Ac denotes the absorbance of CR (without CuONPs) and At denotes the absorbance of the test solution (CR and CuONPs). A blank solution was run in parallel to analyze the degradation of CR under UV light.

## 4. Conclusions

In the present research, CuONPs were synthesised through a greener method and tested against Congo red dye and biological activities. The capping agent on the surface and the CuONP characteristic peaks were confirmed by FTIR analysis. X-ray diffraction showed that the CuONPs were crystalline. The SEM and HRTEM gave information about the structure, shape and size of the CuONPs. The HRTEM images show that CuONPs are small with a large surface area and have a particle size between 25–90 nm. The synthesized particles proved very effective for dye adsorption from polluted water. Moreover, the newly synthesised material showed excellent antibacterial, anti-fungal and antioxidant activities due to its small size, spherical shape and well-dispersed nature. The CuONPs had the highest degradation ability for CR under UV light (catalyst). The H_2_O_2_ further improved the photocatalytic behavior of the CuONPs due to the generation of highly reactive ^•^OH species. The removal of the organic pollutant depended upon the concentration of the oxidants and was stimulated with a higher amount of H_2_O_2_ that initiated the rate of reaction due to a greater inhibition of *e_cb_* -*h_vb_*^+^ recombination and a higher production of the hydroxyl radical. The proposed degradation mechanism for CR chain reactions yielded hydroxyl radical formation and dye degradation resulting in smaller fragments.

## Figures and Tables

**Table 1:** Antibacterial activity of CuONPs.

Bacteria	Clarithromycin	Zone of Inhibition	Inhibition in Percent (%)
*E. coli*	43	15	45
*B. subitilis*	33	13	42
*S. aurous*	38	8	25
*Shigella flexneri*	20	7	34
*Acenotobactor*	29	12	35
*K. pneumonia*	34	----	----
*S. typhi*	40	3	11

**Table 2:** Anti-fungal activity of CuO NPs.

Fungi	Terbinofin (Standard Drug)		@CuONPs	
	Linear Growth (mm)	Inhibition (%)	Linear Growth (mm)	Inhibition (%)
*A. Flavus*	38	62	80	20
*A. niger*	95	5	75	25
*C. albicans*	65	35	100	0
*T. longifusus*	40	60	90	10
*M. Canis*	80	20	100	0
*C. glaberata*	60	40	100	0

**Table 3:** Antioxidant activity against DPPH.

Conc (μM)	Standard Drugs	Concentration-Dependent DPPH% Scavenging
	Ascorbic acid	@CuONPs
25	47.2 ± 0.01	19.2 ± 0.12
50	81 ± 0.05	25.2 ± 0.15
75	129 ± 0.25	50.6 ± 0.04
100	135 ± 0.51	89.2 ± 0.11
(IC_50_, μg/mL)	27 ± 0.02	89.6 ± 0.16

## Data Availability

All the data, including characterizations required for this research are presented in this manuscript.

## Sample Availability

Samples of the compounds are available from the authors.
